# High Seroprevalence against SARS-CoV-2 among Dogs and Cats, Poland, 2021/2022

**DOI:** 10.3390/ani12162016

**Published:** 2022-08-09

**Authors:** Edyta Kaczorek-Łukowska, Kerstin Wernike, Martin Beer, Małgorzata Wróbel, Joanna Małaczewska, Elżbieta Mikulska-Skupień, Karolina Malewska, Izabela Mielczarska, Andrzej Krzysztof Siwicki

**Affiliations:** 1 Department of Microbiology and Clinical Immunology, Faculty of Veterinary Medicine, University of Warmia and Mazury in Olsztyn, Oczapowskiego 13, 10-719 Olsztyn, Poland; 2 Institute of Diagnostic Virology, Friedrich-Loeffler-Institut, Südufer 10, 17493 Greifswald—Insel Riems, Germany; 3 Veterinary Health Center “Lion”, Wilczyńskiego 26, 10-686 Olsztyn, Poland; 4 Veterinary Polyclinic, University of Warmia and Mazury in Olsztyn, Oczapowskiego 14, 10-719 Olsztyn, Poland; 5 Tri-City Veterinary Clinic, Świętokrzyska 33A, 80-041 Gdańsk, Poland

**Keywords:** coronavirus, COVID-19, SARS-CoV-2, cat, dog, prevalence, serology

## Abstract

**Simple Summary:**

The coronavirus SARS-CoV-2 is responsible for the COVID-19 pandemic, which has kept the world in suspense since the beginning of 2020. Infected owners can transmit the virus to their cats and dogs, but it is not exactly known how often this happens. Here, we investigated samples from cats and dogs that live in households in Poland for antibodies against the virus. Antibodies are produced subsequently to a SARS-CoV-2 infection and can be detected for much longer than the virus itself. We found that approximately 18.9% of the cats and 16.0% of the dogs sampled had previously come into contact with the virus and developed antibodies. This suggests a continuous occurrence of virus transmission from infected owners to their pets.

**Abstract:**

The coronavirus SARS-CoV-2 is responsible for a pandemic in the human population that has unfolded since the beginning of 2020 and has led to millions of deaths globally. Apart from humans, SARS-CoV-2 has been confirmed in various animal species, including felines, canines, mustelids, and primates. Of these species, dogs and cats are the most popular companion animals worldwide. Several seroprevalence studies have already been performed in these animal species; however, the results vary depending on the location and especially the time of sampling. Here, serum samples were collected from a total of 388 dogs and 243 cats from three veterinary clinics in two cities (Gdańsk and Olsztyn) in Poland between October 2021 and February 2022, when the country was in the midst of the fourth wave of viral spread. All sera were tested for antibodies against SARS-CoV-2 by a multispecies ELISA based on the receptor-binding domain and by an indirect immunofluorescence assay (iIFA). Overall, 18.9% of the feline sera and 16.0% of the canine sera tested positive using ELISA and iIFA. This relatively high seroprevalence among randomly selected animals is most likely related to the high case numbers in the human population and indicates a continuous occurrence of transspecies virus transmissions from infected owners to their pets. Hence, dogs and cats should be included in monitoring studies and/or outbreak investigations for a better understanding of the epidemiology of this virus.

## 1. Introduction

The viruses of the *Coronaviridae* family are well-known pathogens in both veterinary and human medicine. They are positive-sense, single-stranded, enveloped RNA viruses that are characterized by remarkable genetic plasticity (e.g., point mutations and recombinations) [1,2]. Since the beginning of 2020, a novel member of the coronavirus family has kept the world in suspense, namely, severe acute respiratory syndrome coronavirus 2 (SARS-CoV-2), which is responsible for a pandemic in the human population. The first cases of pneumonia caused by this virus were reported in late 2019 in the Wuhan Province, China [3]. Although it has been more than two years since the first case of SARS-CoV-2 infection in China, there are still open questions about interspecies virus transmissions, intermediate and reservoir hosts, and its effects on animals. SARS-CoV-2 most likely originated from bats, from which it spread to humans, perhaps via one or more intermediate hosts. In the course of the pandemic, natural infections linked to human exposure have been reported in various animal species, including felines, canines, mustelids, and primates [4]. Of these species, dogs and cats are the most popular companion animals worldwide. According to the European Pet Food Industry Federation (FEDIAF), the dog population in Europe in 2020 was over 89 million, while the cat population was as high as 11 million. Increasingly, these pets are friends/family members, meaning that owners are in close contact with them daily, which is important when considering virus transmissions between owners and animals. Indeed, anthropozoonotic SARS-CoV-2 transmissions from infected owners to their cats or dogs were reported with increasing frequency and included multiple variants of concern (VOCs) from alpha to omicron [5,6,7,8,9]. Alarmingly, the suspicion of a first cat-to-human back-transmission of SARS-CoV-2 was reported recently [10]. However, although several studies monitoring infection and seroprevalence among companion animals have been performed worldwide, there are still many uncertainties, such as whether all animals can only be infected by humans, whether intraspecies transmission could also play a role, and what the long-term consequences of passing on the infection to animals could be.

## 2. Materials and Methods

### 2.1. Sample Collection

Serum samples were collected from a total of 388 dogs and 243 cats from three veterinary clinics in two cities (Gdańsk and Olsztyn) in Poland. All of the samples were collected between October 2021 and February 2022 from randomly selected animals that were seen at the clinics (1 in Gdańsk, 2 in Olsztyn) for routine veterinary examinations. Blood samples were collected via forelimb venipuncture, and sera were separated and stored at −20 °C until further analysis. Because the samples tested in this study represented leftover sera from randomized patients, we were only able to obtain information about the health status of both the animal and the owner for a small subset of samples.

### 2.2. Serological Test Systems

All sera were tested for antibodies against SARS-CoV-2 by a multispecies ELISA based on the receptor-binding domain (RBD) performed as described previously [11]. Sera that had a positive or inconclusive reaction were subsequently analysed by an indirect immunofluorescence assay (iIFA) using Vero cells infected with the SARS-CoV-2 strain 2019_nCoV Muc-IMB-1 (multiplicity of infection of 0.1) as an antigen matrix [12,13]. The serum samples were tested in a twofold dilution series starting with 1/8, and FITC-labelled anti-cat IgG (dilution 1/600; Sigma–Aldrich, Steinheim, Germany) and anti-dog IgG (1/100; Sigma–Aldrich) were used as secondary antibodies. The assay was evaluated by fluorescence microscopy.

## 3. Results

Overall, 18.9% (95% confidence interval (CI): 14.0–23.9%) of the feline sera and 16.0% (95% CI: 12.3–20.1%) of the canine sera tested positive/inconclusive by ELISA and positive by iIFA (Table 1 and Table 2). In total, the results of twelve feline sera (4.9%) and 16 canine sera (4.1%) were within the inconclusive measuring range of the RBD-ELISA, and six of these samples per species tested positive using iIFA; these six feline and six canine samples were regarded as seropositive.

From clinic 1 (located in Olsztyn), 48 dogs and 60 cats were tested, and 10.4% (95% CI: 1.8–19.1%) and 15% (95% CI: 6.0–24.0%), respectively, were seroreactive using ELISA and iIFA. Two of the owners indicated that they had been previously infected with SARS-CoV-2 and that they observed clinical signs indicative of a respiratory disease in their animals (Supplementary Tables S1 and S2). A third owner became SARS-CoV-2-infected a month after their animal was sampled; however, it is not known to the authors whether it was its first infection or reinfection by a different virus variant.

In the second clinic (Olsztyn), 30 dogs and 17 cats were sampled. Only dogs (n = 5, 16.7% (95% CI: 3.3–30.0%)) were positive for antibodies to SARS-CoV-2 by ELISA and iIFA (Table 1 and Table 2). All of the seropositive dogs were not regular clients of this clinic and were seen as emergencies or for testing prior to surgery. None of the seropositive animals showed signs characteristic of a SARS-CoV-2 infection when their blood was collected.

Serum samples from 166 cats and 310 dogs were collected from Gdańsk, of which 22.3% (95% CI: 16.0–28.6%) and 16.8% (95% CI: 12.6–20.9%), respectively, reacted positive using ELISA and iIFA. For this clinic, it was not possible to obtain detailed information about the patient’s health status.

## 4. Discussion

Currently, the human SARS-CoV-2 pandemic, with hundreds of millions of infected individuals worldwide, is driven by direct human-to-human virus transmission via aerosolized particles. However, there is increasing evidence of the relevance of animals in SARS-CoV-2 transmission. When the virus was introduced into mink farms, resulting in local epidemics in these highly susceptible animal species, evidence for mink-to-human spillback infections was reported [14], resulting in concerns about viral maintenance in animals. The same holds true for white-tailed deer, for which intraspecies transmission has been reported frequently [15,16,17] and which raised concerns about the establishment of animal reservoirs posing a constant risk for virus back-transmission to humans. Apart from mink and white-tailed deer, SARS-CoV-2 cases have been reported in dogs and cats, among others. Although there have been a number of articles on the seroprevalence of SARS-CoV-2 in companion animals, there are still many unknowns (e.g., regarding their susceptibility to newly emerging VOCs), and the results of the studies vary considerably depending on sample collection time and location [12,18,19,20,21,22,23,24,25].

Here, we serologically investigated cats and dogs in two Polish cities during autumn 2021 and winter 2021/22 and found seroprevalences of 18.9% and 16%, respectively. Earlier during the pandemic, more precisely between June 2020 and February 2021, seroprevalence levels ranging from 1 to 2% were found in dogs and cats in Poland [26]. Such relatively low proportions of seropositive animals (between 0.2% and 6%) have been similarly observed during that time in different countries globally [12,20,21,22,23] and mirrored the situation in the human population. However, as the pandemic progressed and sharp increases in the number of cases were seen in humans worldwide, higher seroprevalences were also observed in companion animals. Patterson et al. 2020 demonstrated a correlation between the number of seropositive animals and the density of SARS-CoV-2 infections in humans [27]. In our study, the samples were collected from October 2021 to February 2022, approximately a year after the first SARS-CoV-2 analysis involving companion animals in Poland. During the sampling period, Poland was in the midst of the fourth wave of the pandemic, with a very high incidence rate in humans. According to the Polish Ministry of Health, in November 2021, the highest incidence rate was recorded in Poland (861 per 100,000 inhabitants), and the dominant virus variant during this period was the delta VOC. Based on the above information, we can conclude that the high seroprevalence observed among dogs and cats was a result of the high number of human infections prior to and during the study period. Studies from Brazil, the US, Canada, and Portugal show that infected owners of dogs and cats were the source of infection for their animals, with higher risks of virus transmission when the dog or cat was in close contact with humans, such as sleeping in the same bed [18,19,24,25]. However, our analyses and specifically the finding of a relatively high seroprevalence could have been biased by the type and location of the sampling veterinarians (=veterinary clinics in two larger cities instead of rural areas) and the selection of animal owners. It could be conceivable that owners that visit veterinarians for routine veterinary examinations, i.e., that are more proactive with veterinary care, could differ in their behaviour towards their animals and their contact frequency and intensities from owners that do not routinely visit veterinarians.

Unfortunately, we do not have any information on the health status of most of the seropositive animals, as the serum samples were randomly collected from patients at the clinics, and the actual time of infection is not known in most cases. Nevertheless, in the small number of animals from which information is available, both the owners and the veterinarians did not observe any concerning clinical signs. Fever, sneezing, and lethargy were only observed in two seropositive cats for a short time, and respiratory signs and/or anxiety were noted in two dogs. One of those dogs exhibited respiratory symptoms for a prolonged period (more than 3 months), but despite that, no changes were visible in the X-ray, and the clinical signs disappeared spontaneously. As the owner was infected with SARS-CoV-2 when the clinical signs were observed, we speculate that the virus was the cause of the disease in the dog. However, direct virus detection by PCR would have been necessary for a final diagnosis, but unfortunately, appropriate swab samples were not available.

In our study, a slightly higher proportion of seropositive animals was found in cats than in dogs (18.9% vs. 16.0%). This finding is consistent with data from both experimental infections and field studies, where cats tend to be more susceptible to the virus than dogs [27,28]. This relatively high susceptibility of cats to SARS-CoV-2 could be related to the high homology of the feline host cell receptor protein angiotensin-converting enzyme 2 (ACE2) to the human orthologue and shows moderate to strong affinity for the S protein of SARS-CoV-2 [29,30]. For dogs, it was discussed that reduced susceptibility to SARS-CoV-2 could be due to low ACE2 expression in the respiratory tract in canines [23]. Nevertheless, although slight differences might exist, both cats and dogs can be infected by SARS-CoV-2 and may shed high amounts of the virus [31,32,33], which could, in rare cases, potentially lead to virus transmission to humans [10]. Hence, dogs and cats should be included in monitoring studies and/or epidemiological investigations, especially when new variants of the virus appear for which the degree of susceptibility of companion animals is unknown. The presence of cats and dogs in such studies will allow for a better understanding of the mechanisms of transmission of SARS-CoV-2 between humans and their pets and will also enable us to notice any change in the effect on animals.

## Figures and Tables

**Table 1:** Number of investigated feline samples and percentage of cats positive for SARS-CoV-2 antibodies by ELISA and iIFA in total and from individual clinics participating in this study.

Veterinary Clinic/City	Number of Cats	Number of Positive Results (%, 95% CI)
1/Olsztyn	60	9 (15.0%, 6.0–24.0%)
2/Olsztyn	17	0 (0%)
3/Gdańsk	166	37 (22.3%, 16.0–28.6%)
Total	243	46 (18.9%, 14.0–23.9%)

**Table 2:** Number of investigated canine samples and percentage of dogs positive for SARS-CoV-2 antibodies by ELISA and iIFA in total and from individual clinics participating in this study.

Veterinary Clinic/City	Number of Dogs	Number of Positive Results (%, 95% CI)
1/Olsztyn	48	5 (10.4%, 1.8–19.1%)
2/Olsztyn	30	5 (16.7%, 3.3–30.0%)
3/Gdańsk	310	52 (16.8%, 12.6–20.9%)
Total	388	62 (16.0%, 12.3–19.6%)

## Data Availability

The data presented in this study are available in the article and its supplementary material.

## Supplementary Materials

The following supporting information can be downloaded at: https://www.mdpi.com/article/10.3390/ani12162016/s1, Table S1: Detailed data obtained about dogs positive for SARS-CoV-2 antibodies (n = 10) from two clinics in Olsztyn; Table S2: Detailed data obtained about cats positive for SARS-CoV-2 antibodies (n = 9) from two clinics in Olsztyn.

## References

[1] Decaro N., Martella V., Saif L.J., Buonavoglia C. (2020). COVID-19 from veterinary medicine and one health perspectives: What animal coronaviruses have taught us. Res. Vet. Sci..

[2] Forni D., Cagliani R., Clerici M., Sironi M. (2017). Molecular evolution of human coronavirus genomes. Trends Microbiol..

[3] Zhu N., Zhang D., Wang W., Li X., Yang B., Song J., Zhao X., Huang B., Shi W., Lu R. (2020). A novel coronavirus from patients with pneumonia in China, 2019. N. Engl. J. Med..

[4] OIE Events in Animals. https://www.oie.int/en/scientific-expertise/specific-information-and-recommendations/questions-and-answers-on-2019novel-coronavirus/events-in-animals/.

[5] Miró G., Regidor-Cerrillo J., Checa R., Diezma-Díaz C., Montoya A., García-Cantalejo J., Botías P., Arroyo J., Ortega-Mora L.M. (2021). SARS-CoV-2 infection in one cat and three dogs living in COVID-19-positive households in Madrid, Spain. Front. Vet. Sci..

[6] Hamer S.A., Ghai R.R., Zecca I.B., Auckland L.D., Roundy C.M., Davila E., Busselman R.E., Tang W., Pauvolid-Correa A., Killian M.L. (2022). SARS-CoV-2 B.1.1.7 variant of concern detected in a pet dog and cat after exposure to a person with COVID-19, USA. Transbound Emerg. Dis..

[7] Jairak W., Chamsai E., Udom K., Charoenkul K., Chaiyawong S., Techakriengkrai N., Tangwangvivat R., Suwannakarn K., Amonsin A. (2022). SARS-CoV-2 delta variant infection in domestic dogs and cats, Thailand. Sci. Rep..

[8] Carneiro R.L., Farias J.P., Pinheiro J.R., Farias J., Vielmo A.C., Birbrair A., Belmok A., Melo F.L., Ribeiro B.M., Chaves G. (2022). First description of a multisystemic and lethal SARS-CoV-2 variant of concern P.1 (Gamma) infection in a FeLV-positive cat. Virol. J..

[9] Sánchez-Morales L., Sánchez-Vizcaíno J.M., Pérez-Sancho M., Domínguez L., Barroso-Arévalo S. (2022). The Omicron (B.1.1.529) SARS-CoV-2 variant of concern also affects companion animals. bioRxiv.

[10] Sila T., Sunghan J., Laochareonsuk W., Surasombatpattana S., Kongkamol C., Ingviya T., Siripaitoon P., Kositpantawong N., Kanchanasuwan S., Hortiwakul T. (2022). Suspected cat-to-human transmission of SARS-CoV-2, Thailand, July-September 2021. Emerg. Infect. Dis..

[11] Wernike K., Aebischer A., Michelitsch A., Hoffmann D., Freuling C., Balkema-Buschmann A., Graaf A., Müller T., Osterrieder N., Rissmann M. (2021). Multi-species ELISA for the detection of antibodies against SARS-CoV-2 in animals. Transbound Emerg. Dis..

[12] Michelitsch A., Hoffmann D., Wernike K., Beer M. (2020). Occurrence of antibodies against SARS-CoV-2 in the domestic cat population of Germany. Vaccines.

[13] Schlottau K., Rissmann M., Graaf A., Schön J., Sehl J., Wylezich C., Höper D., Mettenleiter T.C., Balkema-Buschmann A., Harder T. (2020). SARS-CoV-2 in fruit bats, ferrets, pigs, and chickens: An experimental transmission study. Lancet Microbe..

[14] Oude Munnink B.B., Sikkema R.S., Nieuwenhuijse D.F., Molenaar R.J., Munger E., Molenkamp R., van der Spek A., Tolsma P., Rietveld A., Brouwer M. (2020). Transmission of SARS-CoV-2 on mink farms between humans and mink and back to humans. Science.

[15] Martins M., Boggiatto P.M., Buckley A., Cassmann E.D., Falkenberg S., Caserta L.C., Fernandes M.H.V., Kanipe C., Lager K., Palmer M.V. (2022). From deer-to-deer: SARS-CoV-2 is efficiently transmitted and presents broad tissue tropism and replication sites in white-tailed deer. PLoS Pathog..

[16] Cool K., Gaudreault N.N., Morozov I., Trujillo J.D., Meekins D.A., McDowell C., Carossino M., Bold D., Mitzel D., Kwon T. (2022). Infection and transmission of ancestral SARS-CoV-2 and its alpha variant in pregnant white-tailed deer. Emerg Microbes Infect..

[17] Kuchipudi S.V., Surendran-Nair M., Ruden R.M., Yon M., Nissly R.H., Vandegrift K.J., Nelli R.K., Li L., Jayarao B.M., Maranas C.D. (2022). Multiple spillovers from humans and onward transmission of SARS-CoV-2 in white-tailed deer. Proc. Natl. Acad. Sci. USA.

[18] Barroso R., Vieira-Pires A., Antunes A., Fidalgo-Carvalho I. (2022). Susceptibility of pets to SARS-CoV-2 infection: Lessons from a seroepidemiologic survey of cats and dogs in Portugal. Microorganisms.

[19] Bienzle D., Rousseau J., Marom D., MacNicol J., Jacobson L., Sparling S., Prystajecky N., Fraser E., Weese J.S. (2022). Risk factors for SARS-CoV-2 infection and illness in cats and dogs. Emerg. Infect. Dis..

[20] Michelitsch A., Schön J., Hoffmann D., Beer M., Wernike K. (2021). The second wave of SARS-CoV-2 circulation-antibody detection in the domestic cat population in Germany. Viruses.

[21] Dileepan M., Di D., Huang Q., Ahmed S., Heinrich D., Ly H., Liang Y. (2021). Seroprevalence of SARS-CoV-2 (COVID-19) exposure in pet cats and dogs in Minnesota, USA. Virulence.

[22] Jairak W., Charoenkul K., Chamsai E., Udom K., Chaiyawong S., Hangsawek A., Waenkaew S., Mungaomklang A., Tangwangvivat R., Amonsin A. (2022). Survey of SARS-CoV-2 in dogs and cats in high-risk areas during the second wave of COVID-19 outbreak, Thailand. Zoonoses Public Health.

[23] Ito G., Goto-Koshino Y., Kuroda Y., Eunsil P., Maeda K., Soma T., Momoi Y. (2021). Seroprevalence of antibodies against severe acute respiratory coronavirus 2 (SARS-CoV-2) in household dogs in Japan. J. Vet. Med. Sci..

[24] Goryoka G.W., Cossaboom C.M., Gharpure R., Dawson P., Tansey C., Rossow J., Mrotz V., Rooney J., Torchetti M., Loiacono C.M. (2021). One health investigation of SARS-CoV-2 infection and seropositivity among pets in households with confirmed human COVID-19 cases-Utah and Wisconsin, 2020. Viruses.

[25] Calvet G.A., Pereira S.A., Ogrzewalska M., Pauvolid-Corrêa A., Resende P.C., Tassinari W.S., Costa A.P., Keidel L.O., da Rocha A.S.B., da Silva M.F.B. (2021). Investigation of SARS-CoV-2 infection in dogs and cats of humans diagnosed with COVID-19 in Rio de Janeiro, Brazil. PLoS ONE.

[26] Pomorska-Mól M., Turlewicz-Podbielska H., Gogulski M., Ruszkowski J.J., Kubiak M., Kuriga A., Barket P., Postrzech M. (2021). A cross-sectional retrospective study of SARS-CoV-2 seroprevalence in domestic cats, dogs and rabbits in Poland. BMC Vet. Res..

[27] Patterson E.I., Elia G., Grassi A., Giordano A., Desario C., Medardo M., Smith S.L., Anderson E.R., Prince T., Patterson G.T. (2020). Evidence of exposure to SARS-CoV-2 in cats and dogs from households in Italy. Nat. Commun..

[28] Decaro N., Balboni A., Bertolotti L., Martino P.A., Mazzei M., Mira F., Pagnini U. (2021). SARS-CoV-2 infection in dogs and cats: Facts and speculations. Front. Vet. Sci..

[29] Piplani S., Singh P.K., Winkler D.A., Petrovsky N. (2021). In silico comparison of SARS-CoV-2 spike protein-ACE2 binding affinities across species and implications for virus origin. Sci. Rep..

[30] Guo H., Guo A., Wang C., Yan B., Lu H., Chen H. (2008). Expression of feline angiotensin converting enzyme 2 and its interaction with SARS-CoV S1 protein. Res. Vet. Sci..

[31] Keller M., Hagag I.T., Balzer J., Beyer K., Kersebohm J.C., Sadeghi B., Wernike K., Höper D., Wylezich C., Beer M. (2021). Detection of SARS-CoV-2 variant B.1.1.7 in a cat in Germany. Res. Vet. Sci..

[32] Schulz C., Wylezich C., Wernike K., Gründl M., Dangel A., Baechlein C., Hoffmann D., Röhrs S., Hepner S., Ackermann N. (2021). Prolonged SARS-CoV-2 RNA shedding from therapy cat after cluster outbreak in retirement home. Emerg. Infect. Dis..

[33] Barroso-Arévalo S., Barneto A., Ramos Á.M., Rivera B., Sánchez R., Sánchez-Morales L., Pérez-Sancho M., Buendía A., Ferreras E., Ortiz-Menéndez J.C. (2022). Large-scale study on virological and serological prevalence of SARS-CoV-2 in cats and dogs in Spain. Transbound. Emerg. Dis..

