# Supplementary material for: High Seroprevalence against SARS-CoV-2 among Dogs and Cats, Poland, 2021/2022
Source: Animals (Basel). 2022 Aug 9;12(16):2016. doi: 10.3390/ani12162016 (PMC9404425; doi:10.3390/ani12162016)
Supplement: Supplementary file 1 [file animals-12-02016-s001.zip › animals-1836160-supplementary.pdf]

animals

MDPI

Supplementary Materials

High Seroprevalence against SARS-CoV-2 among Dogs and Cats, Poland, 2021/2022

Supplementary Table S1. Detailed data obtained about dogs positive for SARS-CoV-2 antibodies (n= 10) from two clinics in Olsztyn. IFA – indirect immunofluorescence assay, M – male, F – female.

Numer of dog	Clinic	Gender	Age (years)	Reason for visit of veterinarian	Owner's SARS-CoV-2 health status	Additional information	RBD-ELISA (corr. OD)	iIFA (titer)
1	2	M	9	babesiosis suspected	unknown	none	0.55	1/8
2	2	M	2	blood test prior to ophthalmic surgery - clinically healthy dog	unknown	none	1.21	1/32
3	2	M	2	routine blood test before neutering surgery - clinically healthy	unknown	none	1.597	>1/64
4	2	F	4	bone choking, shallow breathing	became infected a month later with SARS-CoV-2, variant unknown	none	0.239	1/32
5	2	M	2	routine blood test before extraction of a tooth	unknown	none	0.366	1/32
6	1	F	10	persistent cough	owner SARS-CoV-2 confirmed 4-5 weeks before sampling of the dog, variant unknown	serous discharge from the nose and eyes, sneezing, coughing at the time of sampling, clinical signs persisted from December to February 2021, no lung changes on the X-ray, cardiac examination revealed a small mitral valve regurgitation, cough did not respond to antibiotic treatment, gradual improvement after nebulisations, then the clinical signs stopped. At the same time a cat from this household sneezed, but blood samples are not available.	0.252	1/16

7	1	F	5	routine blood tests	none	0.537	1/16
8	1	F	7	routine blood tests	none	0.229	1/16
9	1	M	6	abdominal pain	abdominal pain, and anxiousness	0.671	>1/64
10	1	F	2	routine blood tests	none	0.674	>1/64

Supplementary Table S2. Detailed data obtained about cats positive for SARS-CoV-2 antibodies (n= 9) from two clinics in Olsztyn., M – male, F – female.

Number of cat	Clinic	Gender	Age (years)	Reason for visit of veterinarian	Owner's SARS-CoV-2 health status	Additional information	RBD-ELISA (corr. OD)	iIFA (titer)
1	1	F	5	routine blood tests	unknown	none	1.058	>1/64
2	1	F	9	routine blood tests	unknown	none	1.581	>1/64
3	1	M	6	owners concerned about the cat's behaviour	owner SARS-CoV-2 confirmed three weeks before sampling, variant unknown	fever, sneezing, lethargy observed by the owner during its own illness (clinical signs have begun 3 days after owner's diagnosis)	1.055	>1/64
4	1	M	4	owners concerned about the cat's behaviour	unknown	lack of appetite, fever, lethargy	0.896	>1/64
5	1	M	8	routine blood test	unknown	none	0.355	1/32
6	1	M	11	routine blood test	unknown	none	0.954	>1/64
7	1	M	7	routine blood tests	unknown	none	0.249	1/16
8	1	F	10	routine blood tests	unknown	none	1.535	>1/64
9	1	M	15	routine blood tests	unknown	none	0.449	>1/64
